# *In vivo* evaluation of burn severity in skin tissue of rats using hemoglobin parameters estimated by red–green–blue imaging

**DOI:** 10.1117/1.JBO.30.3.036006

**Published:** 2025-03-24

**Authors:** Rokeya Khatun, Kaisei Okura, Md. Anowar Parvez, Kazuhiro Yashiro, Yuki Nagahama, Yasuyuki Tsunoi, Satoko Kawauchi, Daizoh Saitoh, Shunichi Sato, Izumi Nishidate

**Affiliations:** a Tokyo University of Agriculture and Technology, Graduate School of Bio-Applications and Systems Engineering, Tokyo, Japan; b Tokyo University of Agriculture and Technology, Department of Biomedical Engineering, Tokyo, Japan; c Chittagong Veterinary and Animal Sciences University, Department of Medicine and Surgery, Chittagong, Bangladesh; d National Defense Medical College Research Institute, Division of Bioinformation and Therapeutic Systems, Saitama, Japan; e Kokushikan University, Graduate School of Emergency Medical System, Tokyo, Japan

**Keywords:** tissue oxygen saturation, methemoglobin saturation, total hemoglobin, red–green–blue camera, canonical discriminant analysis, burn severity

## Abstract

**Significance:**

Burn injuries are a global public health problem and are estimated to cause more than 150,000 deaths annually. Even non-fatal burns result in prolonged hospitalization, disfigurement, and disability. The depth of the burn injury is crucial information for selecting adequate treatment for burns. The most common, convenient, and widely used method for assessing burn severity is visual examination, but the accuracy of this method is insufficient, at only 60% to 75%. Rapid and accurate assessment of burn severity is critical for optimal management and treatment of burn patients. Methods of burn severity assessment that are inexpensive, simple, rapid, non-contact, and non-invasive are thus needed.

**Aim:**

We aim to propose an approach to visualize the spatial distribution of burn severity using hemoglobin parameters estimated from a snapshot red–green–blue (RGB) color image and to demonstrate the feasibility of this proposed approach for differentiating burn severity in a rat model of scald burn injury.

**Approach:**

The approach to creating a spatial map of burn severity was based on canonical discriminant analysis (CDA) of total hemoglobin concentration, tissue oxygen saturation, and methemoglobin saturation as estimated from RGB color images. Burns of two different degrees of severity were created in rat dorsal skin by 10-s exposure to water maintained at 70°C and 78°C. RGB color images for the dorsal regions were acquired under anesthesia before burn injury and at 24, 48, and 72 h after injury.

**Results:**

Most areas of images in the groups with skin exposed to 70°C, 78°C, and 37°C water were classified as 70°C burn, 78°C burn, and non-burned normal skin, respectively, over 48 to 72 h. In contrast, classification images of the skin group exposed to 70°C water for 24 h showed a mixture of non-burned normal skin and 70°C burned areas, suggesting that burn severity was heterogeneous.

**Conclusions:**

The proposed approach combining RGB color imaging and CDA appears promising for differentiating 78°C burns from 70°C burns and non-burned normal skin and non-burned normal skin from 70°C and 78°C burns at 24 to 72 h after burn injury in a rat model of scald burn injury.

## 1 Introduction

As one of the most important and common forms of injury worldwide, burns can be caused by heat, chemicals, electricity, and friction. This injury is characterized by prolonged hospitalization, disfigurement, disability, and social rejection. Burns occur more frequently in the least developed and developing countries than in developed countries.^1^^,^^2^ Based on tissue involvement, burns are classified into four degrees. First-degree burns (superficial burns) affect only the epidermis. Second-degree burns also affect the dermis and are further subdivided into superficial dermal burns (SDBs) affecting the epidermis and a small portion of the dermis and deep dermal burns (DDBs) affecting the epidermis and the full thickness of the dermis. Third-degree burns, or deep burns (DBs), result in complete loss of the dermis and necrosis requiring surgical removal.^3^^–^^5^

Rapid, accurate, and continuous assessment of early burn patients is necessary to make final decisions about optimal treatment and management of the injury.^6^ Clinicians and researchers have mainly focused on continuous innovations in modern technologies and methods for rapid and accurate diagnosis of the extent of burns to minimize scar tissue formation and maximize the complete healing process. Nowadays, almost all countries around the world have improved their views, focusing not only on survivability for the patient but also on quality of life and adequacy of healthcare after sustaining burns.^7^ Diagnosing and differentiating between first-degree and third-degree burns is relatively straightforward. The main challenge is seen in the diagnosis of second-degree burns, particularly in differentiating between SDBs and DDBs, as treatment options for these types of burns are markedly different. SDBs require medical and supportive treatment, whereas DDBs require emergency treatment for surgical skin grafting with special care.

The most common and familiar method of burn severity diagnosis is visual and tactile examination by the clinician, but the accuracy is relatively low at ∼71.4%.^8^^,^^9^ Laser Doppler imaging (LDI) methods offer a higher accuracy of ∼97% compared with visual examination.^8^ LDI appears to represent the “gold standard” method for diagnosing burn severity and helps clinicians monitor the healing process of burn injuries.^7^^,^^10^ The main limitations of LDI systems are the cost, and that diagnostic accuracy is only around 80.8% within 24 h and around 92.3% at 72 h after burn injury.^11^ Some researchers have thus recommended that LDI be used between 48 h and 5 days after injury.^12^ Histopathological examinations have also been used to diagnose burn severity, but appear to offer little clinical utility due to the combination of invasiveness, sampling error, and longer processing time.^13^

Optical coherence tomography (OCT) has been applied to evaluate burn depth and morphological and microvascular changes associated with burns have been observed.^14^^–^^16^ Although OCT provides images with high spatial and temporal resolution in a non-contact manner, conventional OCT has relatively low image contrast for different tissues. In addition, applying OCT for future clinical diagnostic use in burns is difficult because of the small field of view and the complexity of analyzing and interpreting OCT images.

Photoacoustic imaging (PAI) is an emerging medical imaging modality that can be used to obtain optical absorption information from deeper tissue layers.^17^^,^^18^ PAI can be applied to evaluate the depth of burn wounds induced at different temperatures,^19^ evaluate burn tissue components such as edema and albumin accumulated in tissues after burn injury,^20^ and monitor changes to the burn wound.^21^ As the photoacoustic wave detector must be positioned on the body surface, PAI is basically a contact measurement.

Spatial frequency domain imaging (SFDI) can estimate concentrations of oxygenated, deoxygenated, and total hemoglobin; oxygen saturation; and collagen content.^22^ Changes in chromophore concentrations after burn injury have been shown to represent the severity of the burn.^23^^–^^25^ Clinical studies have recommended SFDI as a suitable method for differentiating among broad categories of burn severity,^26^ although some limitations remain regarding the very complex analytical methods^27^ with quite dissimilar sensitivity and specificity at 24 h (93% and 74%, respectively) and 72 h (83% and 90%, respectively) after burn injury.^28^

Diffuse reflectance spectral imaging (DRSI) is a promising non-invasive clinical diagnostic technique achieved using simple optical components and devices. DRSI can simultaneously quantify the *in vivo* concentration and oxygen saturation of hemoglobin at each pixel in an image, allowing the assessment of various physiological conditions in living tissues,^5^^,^^6^^,^^24^^–^^30^ and has the potential to be used in evaluating burn severities.^31^^,^^32^ A DRSI method has been developed to spatially map burn severity in skin tissue using canonical discriminant analysis (CDA) with total hemoglobin concentration ($C_{\text{HbT}}$), tissue oxygen saturation (${\mathrm{StO}}_{2}$), and methemoglobin saturation (StMet) estimated from DRSI.^33^ Although DRSI offers high spectral resolution that enables precise spectral analysis, most commercially available spectral cameras are relatively expensive, bulky, and time-consuming to scan a single spectral cube. This represents a problem for clinical application.

Red–green–blue (RGB) color cameras are promising for rapid and cost-effective imaging in burn diagnosis^34^^,^^35^ and have also been widely studied for assessing burn injury. Various approaches based on machine learning techniques for features extractable from burn wounds such as color, shape, texture, and morphology have been proposed to classify burns into those requiring grafting or not,^36^ to assess whether burns would heal spontaneously,^36^ and to classify burns into three depth categories.^37^ With these approaches, concentrations of hemoglobin derivatives represent useful information for assessing burn severity but have not been treated as features entered for machine learning. We have previously proposed a method to quantitatively image biological chromophores in the skin from RGB images.^38^^,^^39^ The present study extended our previously proposed method to evaluate concentrations of oxygenated hemoglobin, deoxygenated hemoglobin, and methemoglobin and proposes a new imaging method for classifying burn injury sites into severity levels based on these three hemoglobin derivatives and estimated saturation values.

## 2 Materials and Methods

### 2.1 Production of Burn Wounds

All experimental procedures were performed in accordance with protocols approved by the Animal Care Committee at the Tokyo University of Agriculture and Technology (Approval Nos. R03–185 and R04–136). Fifteen male Sprague–Dawley albino rats (10 to 12 weeks old; body weight, 210 to 280 g; Tokyo Laboratory Animals Science Co., Tokyo, Japan) were used in this study. Rats were divided into groups with 70°C burns, 78°C burns, and no burns (control), with five rats in each group. Anesthesia was induced in rats with isoflurane (1%, 2 mL/min) and maintained at a depth such that the rat showed no response to toe pinching. After inducing anesthesia, the dorsal and head regions were shaved and a depilatory agent containing thioglycolic acid was applied to both regions. Before producing the burn wound, carprofen (5 mg/kg) was injected intramuscularly for pain relief. Wounds were produced by exposing depilated dorsal skin comprising ∼20% of the total body surface area (4×10  cm) to water maintained at either 70°C, 78°C, or 37°C for the 70°C burns, 78°C burns, and control groups, respectively. Exposure was maintained for 10 s using a Walker–Mason template.^40^ This protocol has been established, and histological examinations have confirmed that burn severities with 70°C burns and 78°C burns correspond to SDB and DDB, respectively.^41^^,^^42^ Immediately after producing the burn, rats were resuscitated with an intraperitoneal injection of saline solution (25 mL/kg). Carprofen (5 mg/kg) was injected intramuscularly twice daily for postoperative pain relief. The wound surface was covered with a moist wound dressing (PA1A; Zuiko Medical Corp., Osaka, Japan) and wrapped in bandages (Daiei Co., Osaka, Japan). Each rat was kept in an independent cage at 50% humidity and a temperature of 24°C with *ad libitum* access to food and water.

### 2.2 Color Image Data Collection

**Fig. 1:**
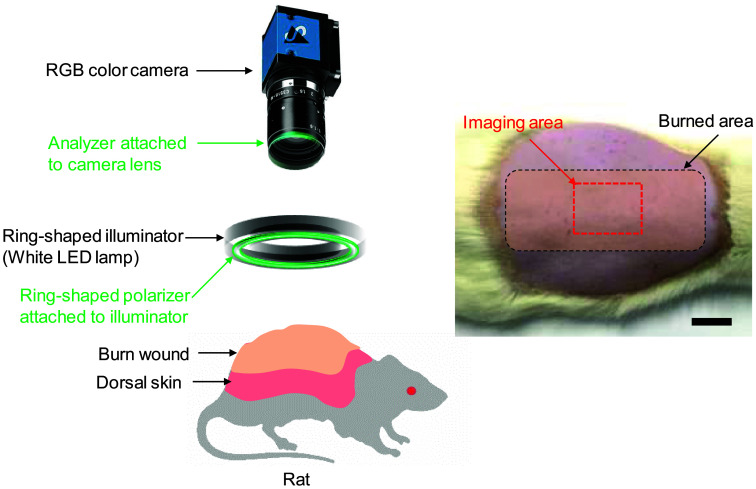
Schematic illustration of the imaging system. The scale bar in the photograph represents 2 cm.

Figure 1 illustrates the experimental apparatus used in this study. A white light–emitting diode light source (LA-HDF158AA; HAYASHI-REPIC Co., Tokyo, Japan) was used to illuminate the sample surface via a light guide with a ring-shaped illuminator. Diffusely reflected light was received by an RGB color camera (DFK21BU618; The Imaging Source, LLC, Charlotte, North Carolina, United States) with a camera lens to acquire an RGB color image. Color image data were captured and recorded on a personal computer. The field of view for the system was an area of $34\times 25\;{\mathrm{mm}}^{2}$ with 640×480  pixels. The lateral resolution of images was estimated to be 53  μm. A standard white diffuser with 99% reflectance (SRS-99-020; Labsphere, North Sutton, New Hampshire, United States) was used to correct for inter-instrument differences in camera output and the spatial non-uniformity of illumination. A ring-shaped polarizer and analyzer were set in a crossed Nicols alignment to reduce specular reflection from the skin surface. The total acquisition time for one image was 0.033 s. Color image data were acquired pre-burn and immediately post-burn under anesthesia. Subsequent measurements were performed at 24, 48, and 72 h after injury. Sequential color images of the dorsal region were acquired from all rats in each group. Euthanasia was the endpoint of the study, just after the collection of the last images. Once sequential data collection had been completed, animals were euthanized by an overdose of isoflurane (5%, 2 mL/min) until 1 min after breathing stopped.

### 2.3 Data Processing for Imaging Hemoglobin Parameters

We had already developed an imaging method for quantifying melanin, oxygenated hemoglobin, deoxygenated hemoglobin, and bilirubin contents in skin tissues.^40^ This method was extended in the current study to evaluate concentrations of methemoglobin, oxygenated hemoglobin, and deoxygenated hemoglobin in the rat model of burn injury. The responses of RGB channels (R, G, and B) in each pixel of the skin tissue color image acquired by a digital RGB camera can be expressed as

$$[\begin{array}{c} R\\ G\\ B \end{array}]=L_{1}[\begin{array}{c} X\\ Y\\ Z \end{array}],$$ (1)

where $L_{1}$ is a transposition matrix to convert tristimulus values X,Y, and Z in the Commission Internationale de l’Éclairage XYZ (CIEXYZ) color system to corresponding R,G, and B responses. Tristimulus values X,Y, and Z in the above equation are defined as

$$X=k\sum E(\lambda )\overset{¯}{x}(\lambda )\Theta (\lambda ),$$ (2)

$$Y=k\sum E(\lambda )\overset{¯}{y}(\lambda )\Theta (\lambda ),$$ (3)

$$Z=k\sum E(\lambda )\overset{¯}{z}(\lambda )\Theta (\lambda ),$$ (4)

where λ, E(λ), and Θ(λ) are the wavelengths, spectral distribution of the illuminant, and diffuse reflectance spectrum of skin tissue, respectively, whereas $\overset{¯}{x}(\lambda )$, $\overset{¯}{y}(\lambda )$, and $\overset{¯}{z}(\lambda )$ are color-matching functions in the CIEXYZ color system. The values of constant *k* that result in Y being equal to 100 for the perfect diffuser are given by

$$k=\frac{100}{\sum E(\lambda )\overset{¯}{y}(\lambda )}.$$ (5)

In Eqs. (2)–(5), the summation can be carried out using data at 10-nm intervals, from 400 to 700 nm. Assuming that the skin tissue mainly comprises an epidermis containing melanin and a dermis containing oxygenated hemoglobin, deoxygenated hemoglobin, and methemoglobin, the diffuse reflectance of skin tissue Θ can be expressed as

$$\begin{array}{l} \Theta =\frac{I}{I_{0}}=[\int_{0}^{\infty }P_{e}(\mu_{s,e}^{\prime },l_{e})\exp(-\mu_{a,m}l_{e})dl]\\ \times [\int_{0}^{\infty }P_{e}(\mu_{s,d}^{\prime },l_{d})\exp[-(\mu_{a,\mathrm{HbO}}+\mu_{a,\mathrm{HbR}}+\mu_{a,\mathrm{metHb}})l_{d}]dl], \end{array}$$ (6)

where I and $I_{0}$ are the detected and incident light intensities, respectively, $P(\mu_{s}^{\prime },l)$ is the path length probability function that depends on the scattering properties as well as on the geometry of measurements, and $\mu_{s}^{\prime }$, $\mu_{a}$, and l are the reduced scattering coefficient, absorption coefficient, and photon path length, respectively. In addition, subscripts m, HbO, HbR, metHb, e, and d indicate melanin, oxygenated hemoglobin, deoxygenated hemoglobin, methemoglobin, epidermis, and dermis, respectively. The absorption coefficient $\mu_{a}$ of each chromophore can be expressed as the product of its concentration C and the extinction coefficient ε and can be defined as

$$\mu_{a}=C\times \varepsilon .$$ (7)

Therefore, the responses of $I_{R}$, $I_{G}$, and $I_{B}$ can be expressed as a function of concentrations of melanin ($C_{m}$), oxygenated hemoglobin ($C_{\text{HbO}}$), deoxyhemoglobin ($C_{\text{HbR}}$), and methemoglobin ($C_{\text{metHb}}$).

**Fig. 2:**
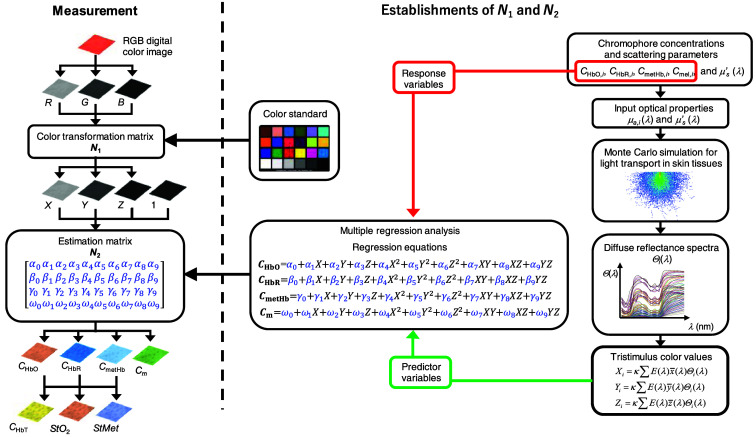
Process for estimating chromophore concentration images from an RGB image.

Figure 2 shows a flowchart describing the estimation process using the proposed method. First, the responses of the RGB channels $I_{R}$, $I_{G}$, and $I_{B}$ in each pixel of the image are transformed into XYZ values by a matrix $N_{1}$ as

$$[\begin{array}{c} X\\ Y\\ Z \end{array}]=N_{1}[\begin{array}{c} R\\ G\\ B \end{array}].$$ (8)

We determined the matrix $N_{1}$ based on measurements of a standard color chart (Color Checker, X-Rite Incorporated, Grand Rapids, Michigan, United States) that has 24 color chips and is supplied with data giving the CIEXYZ values for each chip under specific illuminations and corresponding reflectance spectra. The values of X,Y, and Z are then transformed into $C_{m}$, $C_{\text{HbO}}$, $C_{\text{HbR}}$, and $C_{\text{metHb}}$ by matrix $N_{2}$. Determining matrix $N_{2}$ based on $L_{1}$ and Eqs. (2)–(6) is difficult because $P(\mu_{s}^{\prime },l)$ and l for each layer are usually unknown. We calculated 1550 diffuse reflectance spectra Θ(λ) in a wavelength range from 400 to 700 nm at 10-nm intervals by Monte Carlo simulation (MCS) for light transport^43^ in skin tissue. The simulation model consisted of two layers representing the epidermis and dermis. In a single simulation of diffuse reflectance at each wavelength, 5,000,000 photons were randomly launched. The absorption coefficients of melanin $\mu_{a,m}(\lambda )$, oxygenated hemoglobin $\mu_{a,\mathrm{HbO}}(\lambda )$, deoxygenated hemoglobin $\mu_{a,\;\mathrm{HbR}}(\lambda )$, and methemoglobin $\mu_{a,\mathrm{metHb}}(\lambda )$ were obtained from values of $\varepsilon_{m}(\lambda )$,^44^ $\varepsilon_{\mathrm{HbO}}(\lambda )$,^45^ $\varepsilon_{\mathrm{HbR}}(\lambda )$,^45^ and $\varepsilon_{\mathrm{metHb}}(\lambda )$^46^ in the literature, respectively. The absorption coefficient of the epidermis depends on the volume concentration of melanin in the epidermis, $C_{m}$. We used the absorption coefficient of a melanosome reported in the literature^47^ as the absorption coefficient of melanin $\mu_{a,m}(\lambda )$. This corresponds to the absorption coefficient of the epidermis for the case in which $C_{m}=100\%$. We subsequently derived absorption coefficients of the epidermis for 10 lower concentrations of $C_{m}=1$ to 10% at 1% intervals by making these values simply proportional to that of $C_{m}=100\%$, and absorption coefficients were then input for the epidermis. We assumed the whole blood with 2.32 mM of hemoglobin (corresponding to a hematocrit of 45%) as the 100% volume concentration of total hemoglobin ($C_{\text{HbT}}=100$ vol.%), with uniform distribution in the dermis. The volume concentration in this case represents the percentage of blood in a unit volume of the dermis. Values of $C_{\text{HbO}}$, $C_{\text{HbR}}$, $C_{\text{metHb}}$, and $C_{m}$ were estimated for each pixel. The sum of the values of $C_{\text{HbO}},C_{\text{HbR}}$, and $C_{\text{metHb}}$ represents $C_{\text{HbT}}$. Values of ${\mathrm{StO}}_{2}$ and StMet were calculated as $100\times (C_{\text{HbO}}/C_{\text{HbT}})$ and $100\times (C_{\text{metHb}}/C_{\text{HbT}})$, respectively. Absorption coefficients for total hemoglobin $\mu_{a,\mathrm{HbT}}(\lambda )$ for values of $C_{\text{HbT}}=0.2$ to 1.0 vol.% at 0.2-vol.% intervals were input for the dermis in the MCS. ${\mathrm{StO}}_{2}$ and StMet were determined by $\mu_{a,\mathrm{HbO}}(\lambda )/\mu_{a,\mathrm{HbT}}(\lambda )$ and $\mu_{a,\mathrm{metHb}}(\lambda )/\mu_{a,\mathrm{HbT}}(\lambda )$, respectively, and values ranging from 0% to 100% were used for the simulation. The refractive indices of the epidermis and dermis were assumed to be the same and fixed at 1.4 for all simulations. Thicknesses of the epidermis and dermis were set at 0.06 and 4.94 mm, respectively. The reduced scattering coefficient $\mu_{s}^{\prime }(\lambda )$ derived from the following approximation^48^ was used for both the epidermis and dermis:

$$\mu_{s}^{\prime }(\lambda )=2\times 10^{5}\lambda^{-1.5}+2\times 10^{12}\lambda^{-4}.$$ (9)

XYZ values were then calculated based on the simulated Θ(λ). The above calculations were performed for the various combinations of $C_{\text{HbO}}$, $C_{\text{HbR}}$, $C_{\text{metHb}}$, and $C_{m}$ to obtain datasets of chromophore concentrations and XYZ values. Multiple regression analysis with 1550 datasets established four regression equations for $C_{\text{HbO}}$, $C_{\text{HbR}}$, $C_{\text{metHb}}$, and $C_{m}$

$$C_{\mathrm{HbO}}=\alpha_{0}+\alpha_{1}X+\alpha_{2}Y+\alpha_{3}Z+\alpha_{4}X^{2}+\alpha_{5}Y^{2}+\alpha_{6}Z^{2}+\alpha_{7}XY+\alpha_{8}XZ+\alpha_{9}YZ,$$ (10)

$$C_{\mathrm{HbR}}=\beta_{0}+\beta_{1}X+\beta_{2}Y+\beta_{3}Z+\beta_{4}X^{2}+\beta_{5}Y^{2}+\beta_{6}Z^{2}+\beta_{7}XY+\beta_{8}XZ+\beta_{9}YZ,$$ (11)

$$C_{\mathrm{metHb}}=\gamma_{0}+\gamma_{1}X+\gamma_{2}Y+\gamma_{3}Z+\gamma_{4}X^{2}+\gamma_{5}Y^{2}+\gamma_{6}Z^{2}+\gamma_{7}XY+\gamma_{8}XZ+\gamma_{9}YZ,$$ (12)

$$C_{m}=\omega_{0}+\omega_{1}X+\omega_{2}Y+\omega_{3}Z+\omega_{4}X^{2}+\omega_{5}Y^{2}+\omega_{6}Z^{2}+\omega_{7}XY+\omega_{8}XZ+\omega_{9}YZ.$$ (13)

The regression coefficients $\alpha_{i}$, $\beta_{i}$, $\gamma_{i}$, and $\omega_{i}$ (i=0,1,2, and 3) reflect the contributions of the XYZ values to $C_{\text{HbO}}$, $C_{\text{HbR}}$, $C_{\text{metHb}}$, and $C_{m}$, respectively, and were used as the elements of the 10×4 matrix $N_{2}$ as

$$N_{2}=[\begin{array}{cccccccccc} \alpha_{0} & \alpha_{1} & \alpha_{2} & \alpha_{3} & \alpha_{4} & \alpha_{5} & \alpha_{6} & \alpha_{7} & \alpha_{8} & \alpha_{9}\\ \beta_{0} & \beta_{1} & \beta_{2} & \beta_{3} & \beta_{4} & \beta_{5} & \beta_{6} & \beta_{7} & \beta_{8} & \beta_{9}\\ \gamma_{0} & \gamma_{1} & \gamma_{2} & \gamma_{3} & \gamma_{4} & \gamma_{5} & \gamma_{6} & \gamma_{7} & \gamma_{8} & \gamma_{9}\\ \omega_{0} & \omega_{1} & \omega_{2} & \omega_{3} & \omega_{4} & \omega_{5} & \omega_{6} & \omega_{7} & \omega_{8} & \omega_{9} \end{array}],$$ (14)

Transformation with $N_{2}$ from tristimulus values to chromophore concentrations is thus expressed as

$$[\begin{array}{c} \begin{array}{c} C_{\mathrm{HbO}}\\ C_{\mathrm{HbR}}\\ C_{\mathrm{metHb}} \end{array}\\ C_{m} \end{array}]{=N}_{2}[\begin{array}{c} 1\\ X\\ Y\\ Z\\ X^{2}\\ Y^{2}\\ Z^{2}\\ XY\\ XZ\\ YZ \end{array}].$$ (15)

Once we determine matrices $N_{1}$ and $N_{2}$, images of $C_{\text{HbO}}$, $C_{\text{HbR}}$, $C_{\text{metHb}}$, and $C_{m}$ are reconstructed without the MCS. The total hemoglobin concentration image is simply calculated as $C_{\text{HbT}}=C_{\text{HbO}}+C_{\text{HbR}}+C_{\text{metHb}}$. Images of tissue oxygen saturation and methemoglobin saturation are calculated as ${\mathrm{StO}}_{2}\%=(C_{\text{HbO}}/C_{\text{HbT}})\times 100$ and $\mathrm{StMet}\%=(C_{\text{metHb}}/C_{\text{HbT}})\times 100$, respectively. All image processing was performed using MATLAB version 2018b (MathWorks, South Natick, Massachusetts, United States).

### 2.4 *In Silico* Experiments

We performed *in silico* experiments with diffuse reflectance samples generated by the MCS to validate the accuracy of the proposed method. For test samples, values of $C_{\text{HbT}}$ were set as 0.3, 0.5, and 0.7 vol.%, whereas those of $C_{m}$ were set as 1.5, 3.5, and 5.5 vol.%. Values of ${\mathrm{StO}}_{2}$ ranged from 10 to 89.1%, whereas those of StMet were set to 1, 20, 50, and 80%. Other conditions for the MCS were fixed to be the same as those given in Sec. 2.3. In total, 63 diffuse reflectance spectra at λ=400 to 700 nm in 10-nm intervals were generated under the combinations of $C_{\text{HbT}}$, $C_{m}$, ${\mathrm{StO}}_{2}$, and StMet.

### 2.5 Statistical Analysis

Only one area was used for the dorsal area of each rat for data analysis of color images. A region of interest (ROI) of 300×300  pixels was set at the burn wound area in each image, and the mean and standard deviation (SD) over the ROI were calculated for analyses of the time courses of $C_{\text{HbT}}$, ${\mathrm{StO}}_{2}$, and StMet. Data are thus expressed as mean ± SD. To assess results from *in silico* experiments, Pearson’s correlation coefficient and root mean square error (RMSE) were calculated using ground truth values and estimated values under the proposed method. An unpaired Student’s *t* test was used when comparing among 70°C burn, 78°C burn, and control. A probability value of p<0.05 was taken to indicate statistical significance. CDA was performed to discriminate among 70°C burns, 78°C burns, and no burns (controls) from estimated values of $C_{\text{HbT}}$, ${\mathrm{StO}}_{2}$, and StMet using the proposed method. As the preliminary analysis, we compared the error rate estimates of CDA when using $C_{\text{HbO}}$, $C_{\text{HbR}}$, $C_{\text{metHb}}$, $C_{\text{HbT}}$, ${\mathrm{StO}}_{2}$, and StMet as the predictor variables and those with $C_{\text{HbT}}$, ${\mathrm{StO}}_{2}$, and StMet. As a result, we found that the CDA with $C_{\text{HbT}}$, ${\mathrm{StO}}_{2}$, and StMet has the lower error rate estimates. Therefore, we chose $C_{\text{HbT}}$, ${\mathrm{StO}}_{2}$, and StMet as predictor variables for CDA. In this CDA, time T (s) after burn injury and mean values of $C_{\text{HbT}}$, ${\mathrm{StO}}_{2}$, and StMet over the ROI and their second-order terms were used as predictor variables, whereas the categories of burn severity (i.e., 70°C burn, 78°C burn, and control) were coded as integers and used as response variables. Wilks’ lambda distribution was used to evaluate the significance of separations. The results of discrimination were evaluated by canonical discriminant plots, and the percentage of correct prediction for each sample was evaluated using leave-one-out cross-validation. Discrimination performance was evaluated using a receiver operating characteristic (ROC) curve. The area under the ROC curve (AUC) was calculated to quantify discrimination. When creating the burn severity classification image using leave-one-out cross-validation, one of the 15 rats was used to provide test data, and the remaining 14 rats were used as the training dataset to establish canonical discriminant equations.

### 2.6 Visualizing Spatial Distributions of Burn Severity

**Fig. 3:**
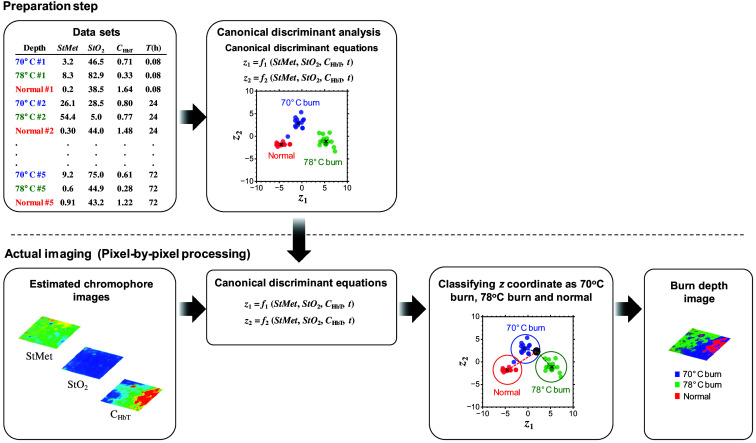
Process of constructing a two-dimensional burn depth classification image.

We proposed a method to visualize the spatial distribution of burn severity based on CDA with images of $C_{\text{HbT}}$, ${\mathrm{StO}}_{2}$, and StMet. Figure 3 shows the process for constructing a two-dimensional burn severity classification image. First, images of $C_{\text{HbT}}$, ${\mathrm{StO}}_{2}$, and StMet are estimated from an RGB image of the dorsal skin of a rat using the method based on multiple regression analysis supported by the MCS.^43^ Applying the canonical discriminant equations established by the CDA described in Sec. 2.4 to each pixel of images for $C_{\text{HbT}}$, ${\mathrm{StO}}_{2}$, and StMet, two-dimensional distributions of canonical scores $z_{1}$ and $z_{2}$ are obtained. Pixel-by-pixel calculations of the distance between a coordinate ($z_{1}$ and$z_{2}$) and the mean value of each burn severity group on the canonical plot are used to finally visualize the spatial map of burn severity.

## 3 Results and Discussion

**Fig. 4:**
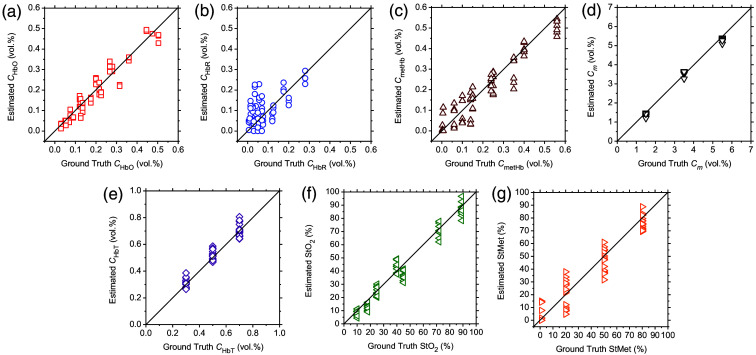
Comparisons between the estimated and ground truth values for $C_{\text{HbO}}$ (a), $C_{\text{HbR}}$ (b), $C_{\text{metHb}}$ (c), $C_{m}$ (d), $C_{\text{HbT}}$ (e), ${\mathrm{StO}}_{2}$ (f), and StMet (g) obtained from the *in silico* experiments.

We validated the accuracy of the method to estimate chromophore concentrations by *in silico* experiments with the XYZ values derived from diffuse reflectance samples generated by the MCS (Fig. 4). Estimated and ground truth values for $C_{\text{HbO}}$, $C_{\text{HbR}}$, $C_{\text{metHb}}$, $C_{m}$, $C_{\text{HbT}}$, ${\mathrm{StO}}_{2}$, and StMet are shown in Figs. 4(a)–4(g), respectively. Values of Pearson’s correlation coefficient between estimated and ground truth values of $C_{\text{HbO}}$, $C_{\text{HbR}}$, $C_{\text{metHb}}$, $C_{m}$, $C_{\text{HbT}}$, ${\mathrm{StO}}_{2}$, and StMet were calculated to be 0.973 (p<0.0001), 0.605 (p<0.0001), 0.949 (p<0.0001), 0.996 (p<0.0001), 0.973 (p<0.0001), 0.982 (p<0.0001), and 0.954 (p<0.0001), respectively. The RMSE of $C_{\text{HbO}}$, $C_{\text{HbR}}$, $C_{\text{metHb}}$, $C_{m}$, $C_{\text{HbT}}$, ${\mathrm{StO}}_{2}$, and StMet were 2.20%, 27.44%, 28.21%, 0.59%, 0.83%, 2.18%, and 24.28%, respectively.

**Fig. 5:**
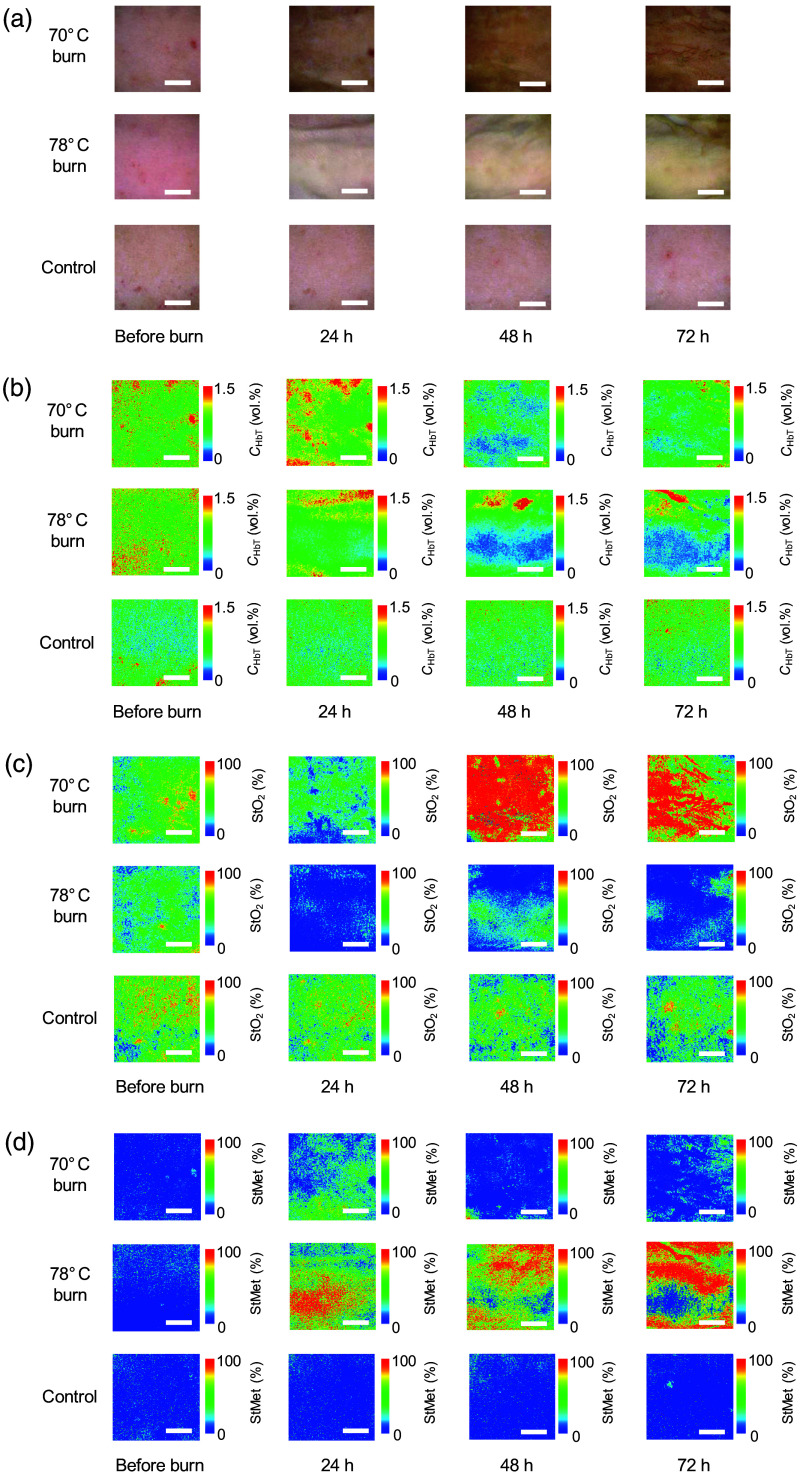
Typical sequential images of RGB color (a), $C_{\text{HbT}}$ (b), ${\mathrm{StO}}_{2}$ (c), and StMet (d) before and after 70°C burn, 78°C burn, and control in rat dorsal skin. The scale bar in each image represents 5 mm.

**Fig. 6:**
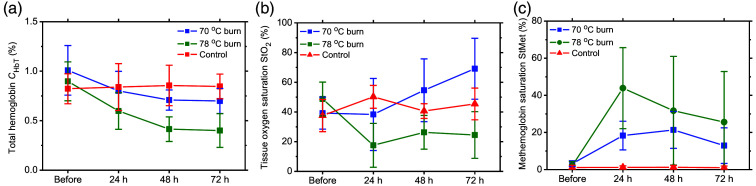
Time courses of $C_{\text{HbT}}$ (a), StO_2_ (b), and StMet (c) before and after 70°C burn, 78°C burn, and control in rat dorsal skin averaged over the ROI of each corresponding image obtained from all samples. Plots and error bars represent the mean and SD of seven rats in each burn injury group, i.e., 70°C burn, 78°C burn, and control in rat dorsal skin.

Figure 5 shows typical sequential images for RGB color, $C_{\text{HbT}}$, ${\mathrm{StO}}_{2}$, and StMet before and after 70°C burn, 78°C burn, and control in rat dorsal skin. Figure 6 shows the time courses of $C_{\text{HbT}}$, ${\mathrm{StO}}_{2}$, and StMet before and after 70°C burn, 78°C burn, and control in rat dorsal skin averaged over the ROI of each corresponding image obtained from all samples. Plots and error bars show the means and SDs for five rats in each group (i.e., 70°C burn, 78°C burn, and control) in rat dorsal skin. In Figs. 5(a) and 6(a), $C_{\text{HbT}}$ in the 70°C burn group showed different time courses from that in the 78°C burn group. In the 70°C burn, $C_{\text{HbT}}$ decreased slightly from 24 to 72 h after burn injury. In contrast, $C_{\text{HbT}}$ in the 78°C burn continued to decrease over time. Total hemoglobin has been observed to be elevated in 70°C burns but stable in 78°C burns.^49^ In thermally injured tissues, microcirculation is progressively disrupted due to tissue destruction, vascular occlusion, microvascular endothelial changes, and thrombus formation. This leads to changes in total hemoglobin concentration in different types of burn injuries. One possible explanation for these changes is venous blood pooling due to impaired vascular supply.

In Figs. 5(b) and 6(b), ${\mathrm{StO}}_{2}$ for 70°C burns was slightly decreased at 24 h then continued to increase at 48 to 72 h. On the other hand, ${\mathrm{StO}}_{2}$ for 78°C burns remained decreased at 24 to 72 h after burn injury. The results at 48 to 72 h for 78°C burns are consistent with findings from other researchers for deep partial-thickness burns, but results for 70°C burns did not match previous findings at 48 to 72 h after burn injury, and ${\mathrm{StO}}_{2}$ for superficial partial burns did not fully recover to pre-burn level.^25^ The estimated values of ${\mathrm{StO}}_{2}$ before burn and no burn skin shown in Fig. 6(b) are in the range of 40% to 50%, which seems low for normal skin. This lower StO_2_ may be related to the fact that the measurements were made under anesthesia and spontaneous breathing, and no thermal blanket was used to maintain normal body temperature.

Figures 5(c) and 6(c) show that StMet for 70°C burns was increased after burn injury and reached a maximum at 24 h after burn injury then closed to pre-burn levels by 72 h after burn injury. In contrast, StMet for 78°C burns was dramatically increased after burn injury and reached the maximum at 24 h after burn injury and then was decreased at 48 and 72 h after burn injury. Time courses for estimated StMet changed depending on burn severity. The mechanisms underlying increased methemoglobin content in burn wounds have been discussed elsewhere.^50^^–^^52^ StMet appears to be one of the most important parameters for burn severity and provides unique ways to assess and characterize different types of burns, and the present findings also suggest that quantifying StMet may provide a critical indicator of the extent of damage within the burn wound.^32^^,^^50^^,^^53^ Methemoglobin is formed by the oxidation of iron moieties in normal hemoglobin from the ferrous state to the ferric state. Levels may thus be increased in injured tissue because of the increased levels of reactive oxygen species generated by neutrophils and reactive nitrogen species (RNS) such as nitric oxide.^52^^,^^54^^–^^56^ Temporal differences in the influx of neutrophils into tissues reportedly exist between superficial and deep burns in human patients.^31^ Oxidation of deoxygenated hemoglobin and oxygenated hemoglobin by RNS also produces methemoglobin.^57^

If some areas of the burn injury created at 78°C unexpectedly reached the tissue below the dermis, which is the depth at which the long-wavelength red light penetrates, it is possible that methemoglobin saturation could approach ∼100%. In addition, burns at 70°C appear to cause relatively little damage to dermal tissue and blood vessels. Therefore, it is possible that most of the methemoglobin produced at the burn site within 24 h disappears by 48 h, resulting in tissue oxygen saturation approaching 100%. Future comparisons with tissue pathology should be made to verify these assumptions.

**Fig. 7:**
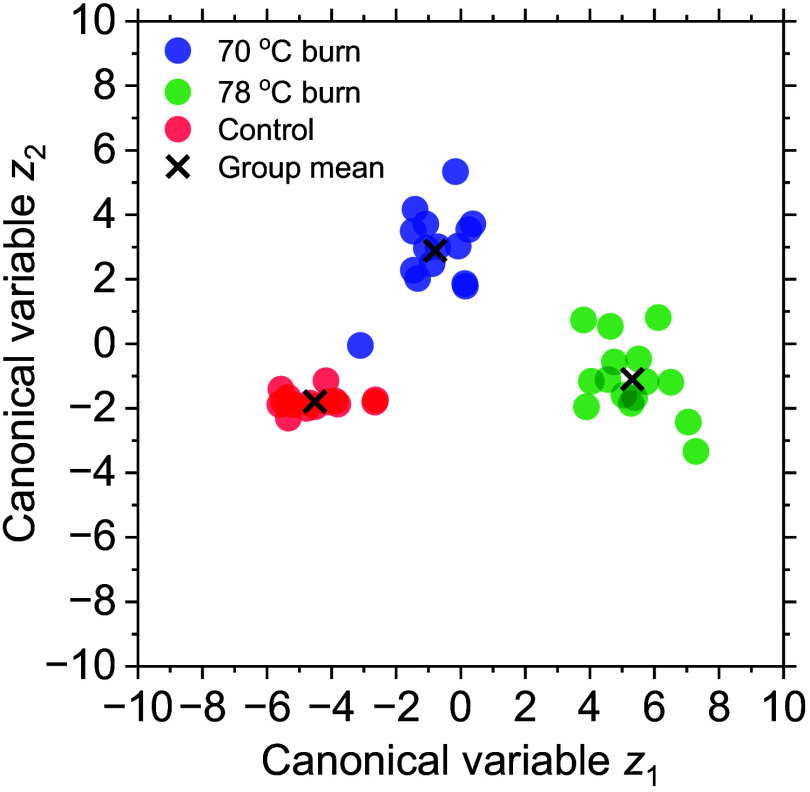
Scatter plots of CDA.

Figure 7 shows the canonical discriminant plots obtained from CDA. These plots showed reasonable separation among 70°C burns, 78°C burns, and controls over time. In this study, 93.33%, 100.00%, and 100.00% of the original grouped cases for 70°C burn, 78°C burn, and control were classified correctly, respectively, compared with 93.33%, 100.00%, and 100.00%, respectively, of cross-validated grouped cases.

**Fig. 8:**
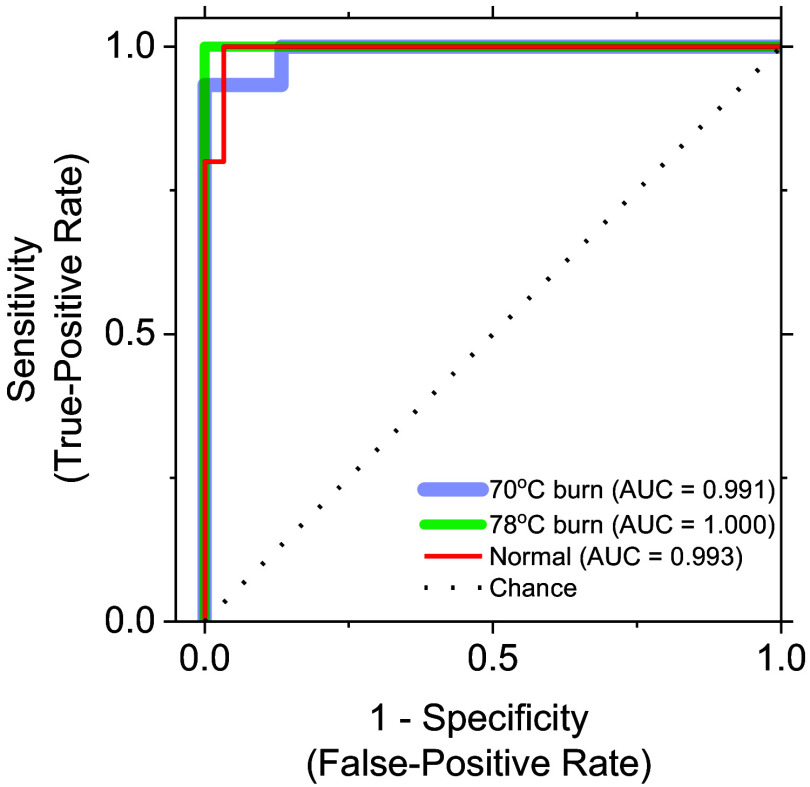
ROC curves for the 70°C burn, 78°C burn, and 98°C burn groups.

Figure 8 shows the ROC curves of the 70°C burn, 78°C burn, and control groups. AUCs for the 70°C burn, 78°C burn, and control groups were 0.993, 1.000, and 0.991, respectively, indicating excellent discrimination accuracy, as expected from the canonical scatterplot results in Fig. 7. Overall, this discriminatory approach using only hemoglobin derivative information appears promising for the classification of 70°C burns, 78°C burns, and control within 72 h after burn injury.

**Fig. 9:**
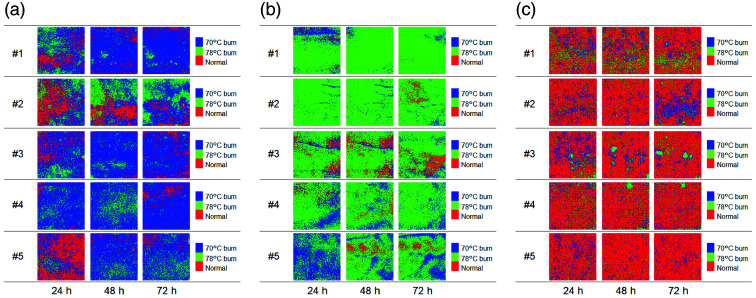
Typical sequential images of burn depth classifications obtained from groups of rats with dorsal skin exposed to 70°C (a), 78°C (b), and 37°C (c) water.

**Fig. 10:**
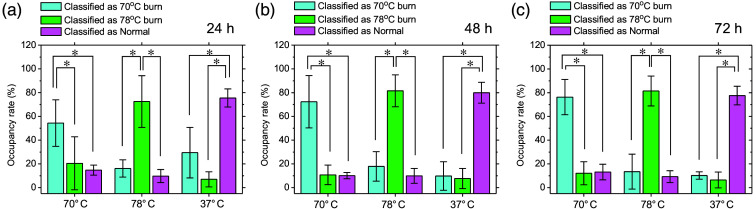
Occupancy rate of pixels classified as 70°C burn, 78°C burn, and control in each image averaged over the four samples for each group at 24 h (a), 48 h (b), and 72 h (c) after burn injury.

Figure 9 shows typical sequential images of burn severity classifications obtained from the groups exposed to 70°C, 78°C, and 37°C water. Figure 10 shows the occupancy rates of pixels classified as 70°C burn, 78°C burn, and control in each image averaged over the five samples for each group at 24, 48, and 72 h after burn injury. For the dorsal skin of rats exposed to 70°C water, areas classified as 70°C burn dominated at 24 to 72 h after burn injury. For the dorsal skin of rats exposed to 78°C water, most areas in burn severity images for all five samples were classified as 78°C burn at 24 to 72 h after burn injury. For the dorsal skin of rats exposed to 37°C water, areas classified as non-burned dominated at 24 to 72 h after exposure to 37°C water.

Burn wounds are not usually uniform in depth, and many comprise a mixture of deep and superficial components.^58^^,^^59^ The thickness of the epidermis and dermis is not strictly constant, but has some variation. In addition, the vasculature within the dermis is also not uniformly distributed. This inhomogeneity in anatomical characteristics could result in spatial variations in heat diffusion within the skin during burn injury and affect the depth distribution of thermal tissue damage. A study using photoacoustic imaging of rat burn wounds induced by the Walker–Mason method showed that the signal representing burn depth was site-dependent, even though uniform heating had been used to create the burn injuries.^41^ This is consistent with the results from burn severity classification images shown in Figs. 9(b) and 10.

Most areas of images from the group with skin exposed to 70°C water were classified as 70°C or 78°C burns, respectively. One possible explanation is that burn severity changed from 70°C burn to 78°C burn because burn wounds are dynamic and can progress as well as convert to deeper wounds.^53^^,^^60^

In this study, 2-dimensional images of burn severity were created based on the results of CDA with the parameters of hemoglobin derivatives. Investigation with CDA showed reasonable results for discriminating among 70°C burn, 78°C burn, and control at 48 to 72 h after burn injury. The method relied on the small training and validation dataset in this study. We therefore performed leave-on-out cross-validation to evaluate correct prediction rates for each sample in CDA with the small dataset.

We also applied a new approach based on CDA for differentiating burn severity. The decomposition of oxygenated hemoglobin and deoxygenated hemoglobin produces not only methemoglobin but also various decomposition products such as bilirubin. Therefore, the absorption spectrum extracted from the diffuse reflectance spectrum of a burn wound becomes complex. However, several research groups have shown that fitting methemoglobin absorption spectra to multispectral and hyperspectral absorption data has advantages in assessing burn severity.^50^^,^^53^ Burn severity images were obtained from the dataset of $C_{\text{HbT}}$, ${\mathrm{StO}}_{2}$, and StMet, allowing 78°C burns and controls to be easily classified but showing a mixture of pixels of all burn severities for 70°C burn at 24 h after burn injury. We used the mean ROI values for $C_{\text{HbT}}$, ${\mathrm{StO}}_{2}$, and StMet as predictive variables to derive the canonical discriminant equations. However, as shown in Fig. 5, $C_{\text{HbT}}$, ${\mathrm{StO}}_{2}$, and StMet displayed spatial heterogeneity. In addition, the mean ROIs for $C_{\text{HbT}}$, ${\mathrm{StO}}_{2}$, and StMet could vary among samples. Although the Walker–Mason method is a well-established protocol for inducing burn wounds with relatively high reproducibility, few studies have investigated the spatial uniformity of burn depths induced by this method. We assumed that burn severities in the dorsal skin of rats exposed to 70°C, 78°C, and 37°C water represented SDB, DDB, and non-burned skin, respectively, based on the literature.^42^ However, some discrepancies may have existed between assumed and actual burn severities for each sample. Determinations of burn depth from histopathological observations of different regions of the burn site at different time points are needed to assign the category names of SDB, DDB, and normal to the response variable in CDA. This should be investigated in future work.

*In silico* experiments used noise-free simulated diffuse reflectance spectral data and corresponding XYZ values to validate the proposed approach. The actual XYZ values calculated from the measured RGB values will contain some noise. Such noisy data may cause variations in the resulting hemoglobin concentrations and their saturation. Therefore, in real cases, the estimated values of each parameter may be worse than *in silico* results.

The present method lacks depth resolution because it relies on integrating all the diffuse reflectance information in the visible wavelength region from 400 to 700 nm along the depth direction, which may represent one factor limiting the accuracy of burn severity classifications. Whether the observed chromophore changes originate from the injured tissue alone or from both injured and underlying non-burned tissues is therefore difficult to determine using this method. We simply selected the central area (300×300  pixels) of the measured image as the ROI. Considering the heterogeneity of estimated images for $C_{\text{HbT}}$, ${\mathrm{StO}}_{2}$, and StMet, differences in the size and position of the ROI seem likely to affect model predictions.

When the proposed method is applied to humans, a large number of $C_{\text{HbT}}$, ${\mathrm{StO}}_{2}$, and StMet data for burn sites of different severity should be collected from human patients and used as a training dataset for CDA. Because albino rats were used in this study, the amount of melanin was close to 0% at all burn severities. In humans, melanin may be present in superficial burns and superficial dermal burns, but with deeper burns, the epidermis may be severely damaged and may slough off. In such cases, no melanin will be present in the burn wound.

In the current study, empirical equations for chromophore concentrations were derived from the MCS with a typical spectrum of light-scattering coefficients. Our current method therefore does not account for variations in the light scattering properties of the skin model. However, this spectrum tends to vary from one part of the body to another and may also vary with the age of the individual. Variability in scattering coefficient spectra may thus affect the accuracy of estimating chromophore concentrations. There may be crosstalk among chromophore estimates because this method attempts to quantify four chromophores based on the three responses of R,G, and B, which are derived from relatively broadband spectra.

Early, accurate assessment of the burn severity is important because the current burn management strategy is early excision and skin grafting of all deep dermal and deep burns.^8^ The current standard waiting time for a decision on grafting or continued wound care is 3 to 7 days after the burn.^25^ LDI is the only technique approved by the Food and Drug Administration for burn assessment. This method has been shown to reduce surgical workload by eliminating unnecessary surgeries and is used to predict burn healing between 48 h and 5 days post-burn.^9^ Our proposed approach with CDA for $C_{\text{HbT}}$, ${\mathrm{StO}}_{2}$, and StMet in burn wounds showed reasonable results for discriminating among 70°C burns, 78°C burns, and normal at 24 to 72 h after burn injury. The proposed method can therefore meet the clinical need for diagnosis after injury and for use in decision-making regarding grafting or continuation of burn wound care.

We have previously reported the method to spatially map burn severity in skin tissue using CDA with $C_{\text{HbT}}$, ${\mathrm{StO}}_{2}$, and StMet estimated from diffuse reflectance spectral images.^33^ The previous study used the same methodology as the present study, except that images were taken with a hyperspectral camera instead of an RGB camera, and CDA was performed on 70°C, 78°C, and 98°C burns. The time courses of $C_{\text{HbT}}$, ${\mathrm{StO}}_{2}$, and StMet for both 78°C burns and no burns after burn injury obtained in this study showed similar trends to those obtained in the previous study. On the other hand, there were slight differences in the time courses of $C_{\text{HbT}}$, ${\mathrm{StO}}_{2}$, and StMet for 70°C burns between the two studies. In this study, CDA was performed on a dataset consisting of no burns, 70°C burns, and 78°C burns, whereas the previous study using hyperspectral imaging investigated 70°C, 78°C, and 98°C burns. Therefore, the performance of the two CDAs should be carefully compared. In both studies, there was good separation of 70°C and 78°C burns in the discriminant plot results. The present study also showed good separation of 70°C, 78°C, and no burn injuries. On the other hand, the previous study showed that the distributions for 78°C and 98°C burns tended to overlap somewhat in the scatter plot of the CDA, resulting in a relatively low AUC of the ROC curve for 78°C burns. It would be expected that discrimination performance would deteriorate if the present method using an RGB camera were used to discriminate among 70°C, 78°C, and 98°C burns. For a more detailed performance comparison of the two approaches, comparative studies using a common dataset of non-burns and 70°C, 78°C, and 98°C burns should be conducted in the future.

We created a burn wound on a large area of the dorsal skin of rats. Each image shown in Figs. 5 and 9 corresponds to an area of $1.59\times 1.59\;{\mathrm{cm}}^{2}$ around the center of the burn wound. Because this entire is considered to be a burn wound, it is difficult to see the boundary between burned and unburned areas in Figs. 5 and 9. Imaging of burns created with a heated brass comb^6^ would be extremely useful for better analysis of burn severity discrimination performance at the boundary between burned and unburned areas.

The sampling depth of the method is related to the depth of light penetration in the entire visible wavelength range of 400 to 700 nm, as the method is based on the reflected light intensities of the red, green, and blue channels. Penetration depth is defined by the absorption and scattering coefficients of the target tissue, which vary with wavelength. In addition, the absorption coefficient of the tissue also depends on the amount of chromophores present. Our results showed that the amount of each chromophore varies depending on the severity of the burn injury. In DDBs and DBs, scattering coefficients reportedly decrease significantly after burn injury.^25^ Therefore, it should be noted that the sampling depth of this method may vary depending on the severity of the burn injury. The 5-mm skin thickness of the MCS in this study does not resemble the actual rat skin thickness. To better mimic rat skin, the second layer representing the dermis in the MCS should be semi-infinite in thickness. Although the diffuse reflectance spectrum obtained from the MCS model with the semi-infinite thickness of the second layer is not significantly different in the short and medium wavelength region from that obtained from the MCS model used in this study, the reflectance is expected to be high in the long wavelength region. Such a difference in reflectance will be reflected primarily in the response of the red channel. This results in different estimates of oxy-, deoxy-, and methemoglobin amounts and saturations. The impact of differences in MCS modeling on the results of burn severity classification should be investigated in future work.

## 4 Conclusions

The present study demonstrated a method for classification and imaging of burn severity in rats based on the combination of RGB color imaging and CDA. ROC curves and AUC values indicated excellent discrimination accuracy. The proposed approach with CDA for ${\mathrm{StO}}_{2}$, StMet, and $C_{\text{HbT}}$ in burn wounds showed reasonable results for discriminating among 70°C burns, 78°C burns, and non-burned normal skin at 48 to 72 h after burn injury.

## Data Availability

Data underlying the results presented in this paper are not publicly available at this time but may be obtained from the authors upon reasonable request and through a collaboration agreement.
